# Large Lung Consolidation: A Rare Presentation of Pulmonary Sarcoidosis

**DOI:** 10.3390/life14010044

**Published:** 2023-12-27

**Authors:** Monica Steluta Marc, Camelia Corina Pescaru, Emanuela Oana Costin, Alexandru Florian Crisan, Adelina Maritescu, Andrei Pescaru, Noemi Suppini, Gheorghe Emilian Olteanu, Daniel Traila, Cristian Oancea, Diana Manolescu

**Affiliations:** 1 Center for Research and Innovation in Precision Medicine of Respiratory Diseases, “Victor Babes” University of Medicine and Pharmacy, Eftimie Murgu Square 2, 300041 Timisoara, Romania; marc.monica@umft.ro (M.S.M.); noemi.suppini@umft.ro (N.S.); olteanu.gheorghe@umft.ro (G.E.O.); traila.daniel@umft.ro (D.T.); oancea@umft.ro (C.O.); 2 Pulmonology Clinic, Clinical Hospital of Infectious Diseases and Pulmonology, “Victor Babes”, Gheorghe Adam Street 13, 300310 Timisoara, Romania; emanuelalucaci0509@gmail.com (E.O.C.); crisan@umft.ro (A.F.C.); adelina.maritescu@umft.ro (A.M.); dmanolescu@umft.ro (D.M.); 3 Connolly Hospital Blanchardstown, Mill Road, Abbottstown, D15 X40D Dublin, Ireland; 4 Research Center for the Assessment of Human Motion, Functionality and Disability (CEMFD), “Victor Babes” University of Medicine and Pharmacy, Eftimie Murgu Square 2, 300041 Timisoara, Romania; 5 Doctoral School, “Victor Babes” University of Medicine and Pharmacy, Eftimie Murgu Square 2, 300041 Timisoara, Romania; 6 Faculty of General Medicine, “Victor Babes” University of Medicine and Pharmacy, Eftimie Murgu Square 2, 300041 Timisoara, Romania; andrei.pescaru@student.umft.ro; 7 Center of Expertise for Rare Lung Diseases, Clinical Hospital of Infectious Diseases and Pneumophthisiology “Dr. Victor Babes” Timisoara, 300041 Timisoara, Romania; 8 Research Center for Pharmaco-Toxicological Evaluations, Faculty of Pharmacy, “Victor Babes” University of Medicine and Pharmacy, 300041 Timisoara, Romania; 9 Department of Radiology and Medical Imaging, “Victor Babes” University of Medicine and Pharmacy, 300041 Timisoara, Romania

**Keywords:** pulmonary sarcoidosis, diagnosis, treatment

## Abstract

Sarcoidosis is a complex inflammatory disease of uncertain origin, characterized by non-necrotizing epithelioid cell granulomas (NNEGs) affecting multiple organ systems. Although many different clinical and pathological phenotypes can be present, with different organs involved, the lung is the most common site described. In this case report, we (a) present and discuss the broad differential diagnosis of a patient presenting with a solitary lung mass with clinical and imaging features of lung cancer that ultimately was confirmed with a rare manifestation of stage II pulmonary sarcoidosis, and (b) analyze and compare similar cases from the literature.

## 1. Introduction

Sarcoidosis is currently described as a multifaceted disease with a multisystem inflammatory disease of unknown cause, characterized by multiple granulomas in every organ involved in the disease process, with the lungs being the most common site of involvement [1]. The incidence and prevalence of sarcoidosis vary greatly based on region, sex, race, and age groups; notwithstanding these variables, men are generally more affected than women at a younger age [1,2]. The prevalence of involvement of the lungs in sarcoidosis is between 89–99%, with major related symptoms like increased fatigue, general malaise, dyspnea, cough, wheezing, and stridor. There is a high risk of developing lung fibrosis and irreversible damage to other organs, like the lymph nodes, the heart, the skin, the eyes, the liver, the kidneys, or the neurological system. Löfgren syndrome, a specific acute clinical manifestation of sarcoidosis, manifests with a triad of fevers, erythema nodosum, and bilateral hilar lymphadenopathy on chest radiography. Because of the non-specific symptoms of this syndrome, computed tomography (CT) scans play a crucial role in making a diagnosis [3,4]. Common findings include bilateral, symmetrical micronodules in a peribronchovascular distribution with upper and middle lung predominance, accompanied by bilateral, symmetrical hilar lymphadenopathy [5]. From a diagnostic point of view, the atypical radiographic patterns of pulmonary sarcoidosis are complicated, especially in rare cases of mass lesions, because the associated symptoms can mimic a malignancy [6].

The objectives of our study are to (a) explore the differential diagnosis of a solitary lung mass resembling lung cancer, ultimately confirmed as a rare manifestation of stage II pulmonary sarcoidosis, and (b) analyze and compare similar cases from the literature to enhance understanding of the presentation, diagnosis, and management of pulmonary sarcoidosis masquerading as lung cancer.

## 2. Case Presentation

A 72-year-old female, never smoker, with type 2 diabetes, arterial hypertension class II, and obesity, presented with cough with intermittent sputum, dyspnea on exertion and inspiratory wheezing, weight loss (approximately 3 kg in the previous month), and fatigue.

The clinical examination revealed bilateral wheezing crackles in both lung areas and an oxygen saturation level of 90% in ambient air. The spirometry test was significant for moderate obstructive pulmonary dysfunction.

### 2.1. Laboratory, Radiological, and Histological Examinations

**Figure 1:**
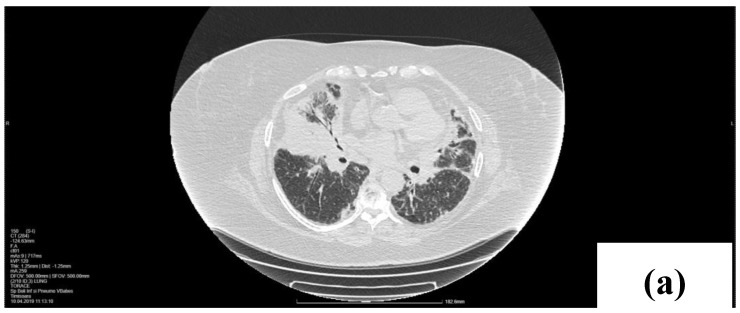

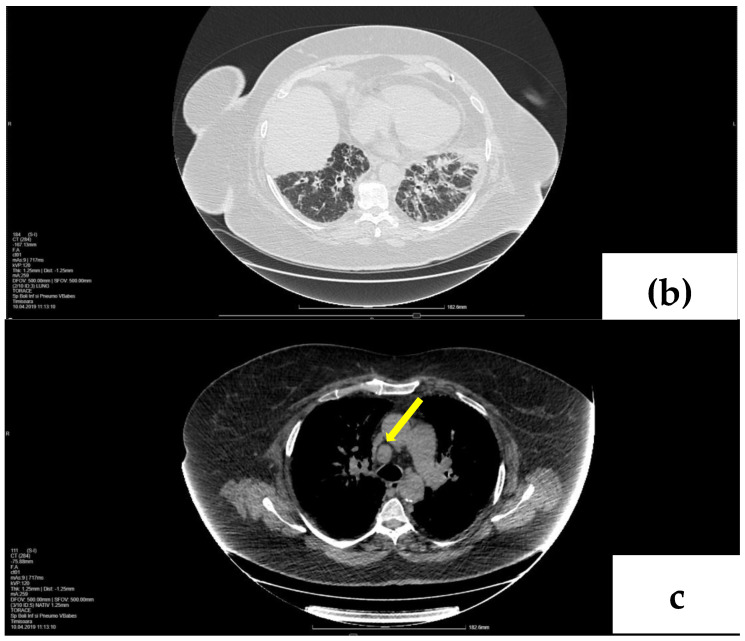
CT scan at the baseline. Axial lung windows at the level of the middle lobe (**a**) and lower lobes (**b**) with a dense mass in the right middle lobe (RML), cuffing of bronchovascular bundle with perilymphatic micronodules. (**c**) Right paratracheal (4R) lymphadenopathy (arrow).

A lesion resembling a tumor situated in the right middle lobe (RML), encircling the origin of the lobular bronchus, was revealed during the chest computed tomography (CT). We observed a bilateral thickening of the pulmonary perilymphatic space in the upper and lower lobes, peribronchial cuffing, and subpleural consolidations in the posterior lower lobes. Right paratracheal and subcarinal lymph node enlargement could be seen in the mediastinal window (Figure 1).

The initial blood tests revealed inflammatory parameters and serum glucose with high values. The autoantibody screens for the antinuclear antibody (ANA) and antineutrophil cytoplasmic antibodies (ANCAs) were negative.

The medical staff performed a bronchoscopy after conducting a CT examination to further investigate the patient’s condition. An almost complete obstruction of the right middle lobe bronchus, with an infiltrative appearance, was noticed, leading to a biopsy performed at this level. The histopathological aspect of the successive sections advocated for a chronic granulomatous inflammatory process with multinucleated giant cells.

**Figure 2:**
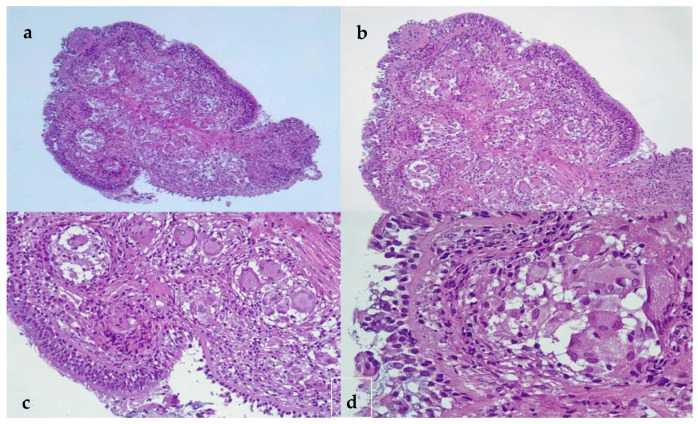
Histopathology of the bronchial biopsy specimen (**a**) ×40 HE, (**b**) ×100 HE, (**c**) ×200, (**d**) and ×400. Nodular lesions along the bronchial wall (polypoid-like appearance) with non-necrotizing epithelioid granulomas with inflammatory lymphoplasmacytic infiltrate. Langhans-type multinucleated giant cell with Schaumann body. HE-hematoxylin and eosin.

A multidisciplinary consensus suggested a second biopsy because of the discrepancy between the CT and bronchoscopy appearance of the infiltrative mass and the histological pattern of sarcoidosis. The patient revisited the clinic two weeks post-discharge, experiencing a decline in her overall health status. After the second bronchoscopy, the histological examination showed no characteristic or suggestive signs to support a malignancy diagnosis. All histopathology aspects advocated for a specific chronic granulomatous inflammation (giant epithelioid granulomas), confirming the diagnosis of sarcoidosis (Figure 2).

### 2.2. Treatment and Outcome

The clinical and laboratory investigations, particularly the two histopathological findings, disproved our initial suspicion of a proliferative process in the right middle lobe (RML) and provided evidence of an atypical aspect supporting the diagnosis of sarcoidosis. At discharge, the patient received treatment with an oral systemic corticosteroid, Prednisone, 40 mg/day.

**Figure 3:**
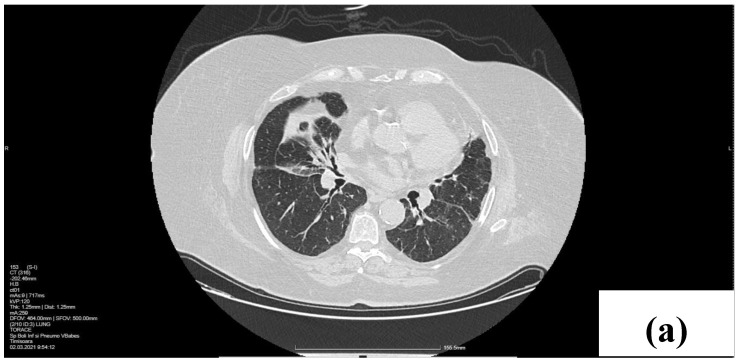

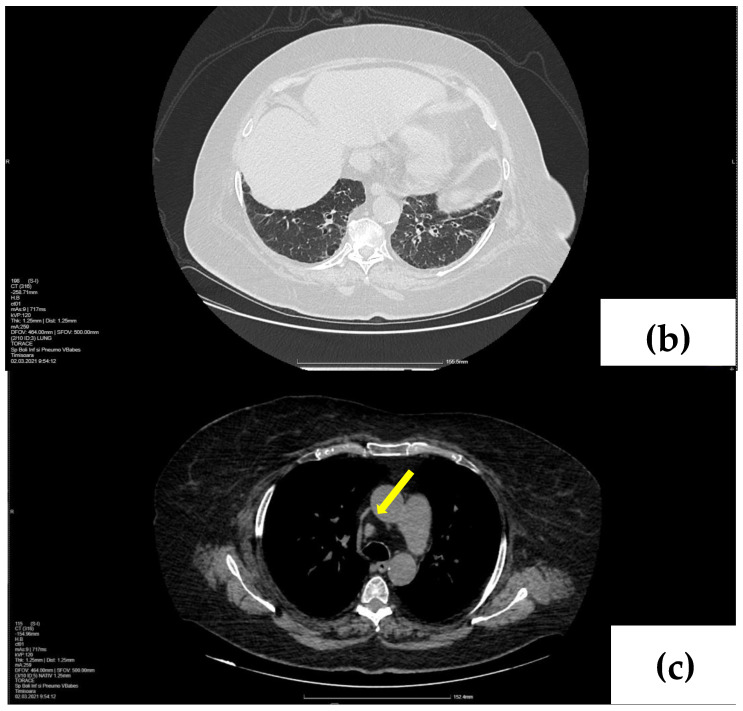
CT scan after treatment. Axial lung windows at the level of the middle lobe (**a**) and lower lobes (**b**) show a decrease in the RML mass and some scars in the lower lobes. (**c**) Right paratracheal (4R) lymphadenopathy involution (arrow).

The patient was evaluated three months after treatment initiation. The clinical status improved, and the CT findings also supported this. A significant regression of the RML lung mass was noticed, only with some fibrotic scars along the bronchovascular bundle. Also, an involution of mediastinal adenopathy was observed (Figure 3). The pulmonary sarcoid lesions had entered a state of remission due to oral systemic corticotherapy, and we advised a tapering of the Prednisone dosage up to 5 mg/day with the treatment of concurrent health conditions.

## 3. Discussion

Sarcoidosis is a multifaceted, multisystemic inflammatory disease with an unclear etiology, characterized by non-necrotizing granulomas. Despite its long history, with the first case described in 1877 by Jonathan Hutchinson in London, this pathology has remained mysterious [2]. Its unidentified cause and multisystemic spread have made it more challenging and complex, and, therefore, the comprehension of sarcoidosis and its diagnosis has never been fully secured [2]. Even though there is no standard diagnosis of sarcoidosis, the American Thoracic Society recommends three essential criteria to look for: a susceptible clinical presentation, non-necrotizing granulomas in tissue biopsy, and the consideration of a differential diagnosis for granulomatous disease. The clinical manifestations may vary depending on the severity of the disease and multiple organ involvement. In most cases, the disease is asymptomatic. However, there could be non-specific and negligible symptoms such as fever, dry cough, breathlessness, or chest discomfort. The most common systemic symptoms are prolonged tiredness, night sweats, and weight loss [7].

Sarcoidosis may have an acute, sub-acute, or chronic clinical presentation. Healthcare providers should always consider some highly specific clinical features despite the challenging nature of diagnosing sarcoidosis. These include Lofgren syndrome, Heerfordt–Waldenstrom syndrome, and lupus pernio (a disorder affecting the skin and internal organs). Heerfordt–Waldenstrom syndrome is a rare sarcoidosis variant characterized by parotid gland enlargement, facial nerve palsy, anterior uveitis, and fever. However, Lofgren syndrome, characterized by fever, erythema nodes, and bilateral hilar adenopathy, is one of the most common presentations of acute sarcoidosis.

Due to the non-specific clinical manifestations of sarcoidosis, the histopathological demonstration of non-necrotizing granulomas is often necessary for an accurate diagnosis. The accuracy of the diagnosis involves a sensible differential for alternative diagnoses of infectious or non-infectious causes since the result of the histopathological examinations found in patients with sarcoidosis has no unique features to differentiate it from other granulomatous diseases. Thus, additional screenings for active or latent tuberculosis infection, tissue culture, bronchoalveolar lavage (BAL), or serological tests for fungal infections may be conducted to support a differential diagnosis [8,9].

While research has identified potential sarcoidosis biomarkers in peripheral blood lymphocytes and BAL fluid, identifying differentially expressed miRNAs through tissue biopsies remains limited. A considerable portion of miRNA studies have involved peripheral blood mononuclear cells (PBMCs) and lung tissue samples, raising the possibility of overlapping miRNA expression profiles between these two sources [10].

Due to their remarkable stability in human fluids, circulating microRNAs hold immense promise as diagnostic and prognostic biomarkers for various diseases. Moreover, transforming miRNA-based therapies from research laboratories to clinical practice holds tremendous potential for revolutionizing healthcare [11].

Dylan Kelleher et al. reported that researchers rarely report a large, solitary pulmonary mass, and its incidence is unknown, possibly arising from the coalescence of individual granulomas to create the appearance of a mass [12].

Mulkareddy et al. presented a similar case, reporting a 39-year-old African American male who sought medical attention due to a persistent cough and sharp chest pain exacerbated by breathing. Initial diagnostic imaging identified an abnormal opacity in the right lower lobe of the lung, raising concerns about potential pneumonia. Despite receiving a course of antibiotics, both the patient’s symptoms and the radiological findings remained unchanged. Subsequent PET scanning showed increased fluorodeoxyglucose (FDG) uptake in the right lower lobe, suggesting a pathological pulmonary mass.

A biopsy of the pulmonary mass and associated lymph nodes revealed results that indicated non-caseating granulomas, a characteristic feature of sarcoidosis [13].

This unique case highlights an uncommon presentation of pulmonary nodular sarcoidosis, which typically appears as diffuse infiltrates rather than a solitary mass.

In our case, a bronchoscopy and histopathology exam confirmed the diagnosis. The main characteristic of a sarcoidosis granuloma is the concentrically round shape of immune cells arranged in layers, with the prevalence of a central core made of macrophages and multinucleated giant cells. T lymphocyte cells are usually found in the outer layer, with interposed dendritic cells and sometimes a few isolated collections of B lymphocytes [8,14].

The presence of sarcoid-like granulomas can sometimes occur concurrently with or appear after a cancer diagnosis. Detecting granulomas in a biopsy of mediastinal lymph nodes in a patient with pulmonary nodules raises the concern of potential undiagnosed lung cancer [15].

The authors discuss the case of a 71-year-old man with a history of chronic obstructive lung disease (COPD) who initially presented with a 9.9 mm lung nodule and mediastinal lymphadenopathies. An initial endobronchial ultrasound-guided transbronchial fine-needle aspiration (EBUS-TBNA) of lymph nodes in stations 7 and 4R showed normal lymph node structure. Two years later, a surveillance chest CT scan revealed nodule growth to 15 mm, with PET/CT showing FDG-avid nodules and mediastinal lymph nodes. He experienced shortness of breath during exertion and had quit smoking after a 50-pack-year history. Subsequent EBUS revealed non-necrotizing granulomas in the 4L and 11L lymph nodes, leading to a referral for sarcoidosis treatment. A biopsy of the nodule was also conducted due to concerns of cancer sarcoid syndrome, ultimately confirming a poorly differentiated lung adenocarcinoma positive for GATA3, P63, CK7, and TTF-1 [15].

Inversely, there was a case report of a delayed diagnosis of lung cancer due to a misdiagnosis as worsening of sarcoidosis. The authors presented a case of a 68-year-old man initially with a mass in the right hilar region and lymphadenopathy in the subcarinal and bilateral interlobar areas on chest CT. Subsequent endobronchial ultrasonography (EBUS)-guided transbronchial needle aspiration (TBNA) provided core samples from the subcarinal and bilateral interlobar lymph nodes, confirming a diagnosis of sarcoidosis. However, after 5 months, his condition worsened with the development of right upper paratracheal lymphadenopathy. Initially treated as an exacerbation of sarcoidosis with oral corticosteroids, a follow-up CT scan revealed new right lower paratracheal lymphadenopathy and worsening right hilar lymphadenopathy. A subsequent bronchoscopy and EBUS procedure led to the discovery of adenocarcinoma from the lung in a transbronchial lung biopsy and EBUS-TBNA from the right lower paratracheal lymph node [16].

Mehta presented the case of a 54-year-old woman referred to a pulmonary clinic with suspicions of malignancy. They performed a right thoracotomy, including a right upper lobe wedge resection and a partial mediastinal lymphadenectomy. The pathology results indicated localized organizing pneumonia with associated giant-cell reaction and non-necrotizing granulomas with focal peribronchiolar hyaline fibrosis. An excised right paratracheal lymph node showed an anthracitic lymph node with hyalinized granulomas. The microbiological tests were negative for tuberculosis and fungal infections. Additional screenings for autoimmune diseases and fungal infections were also negative. A confirmation of the diagnosis of pulmonary sarcoidosis as a solitary pulmonary nodule was concluded. It was identified that there was no extrapulmonary disease or other pulmonary involvement, and the patient recovered without needing further treatment [17].

Nodular sarcoidosis is a rare variant of sarcoidosis, accounting for approximately 2.4–4% of cases. Typically, patients exhibit multiple lung nodules, while a solitary lung nodule is uncommon, occurring in around 18% of nodular sarcoidosis cases. Distinguishing between malignancy and nodular sarcoidosis based on radiological features is challenging, highlighting the importance of histological examination to achieve a precise diagnosis [18].

Taketa et al. introduced a 67-year-old woman with no significant medical history and a non-smoking background who presented to the hospital following an abnormal chest radiograph finding. A physical examination revealed no immediate distress, and the laboratory tests showed slightly elevated C-reactive protein levels. Lung cancer markers and angiotensin-converting enzymes were within normal ranges. A computed tomography (CT) scan revealed a solitary nodule with speculations in the right lung’s S6. A positron emission tomography (PET)/CT scan indicated mild fluorodeoxyglucose uptake in the nodule and the right hilar lymph node. A transbronchial biopsy did not reveal any malignant findings. Subsequently, video-assisted thoracoscopic lung surgery on the right lower lobe nodule confirmed non-caseating granulomas consistent with sarcoidosis. Acid-fast bacilli stains and tissue cultures were negative, solidifying the diagnosis of sarcoidosis [19].

Since many organs can be targets of the disease, the imagining technique plays an essential role in its diagnosis and location [20]. Even though, in up to 90% of cases, the lungs and intrathoracic lymph nodes are the most frequently involved locations, the radiological patterns may sometimes be non-specific [21]. The most common HRCT pictures, classified as stages 0–4 in the Scadding classification, can sometimes be hidden by unusual appearances. In an appropriate clinical presentation, the pattern exhibits parenchymal and mediastinal involvement, where lymphadenopathy is mostly present at the carinal, subcarinal, and hilar levels, associated with typical perilymphatic node distribution [22].

This selected predilection allows the grouping of the nodules along the bronchial bundles, interlobular septa and fissures, and subpleural zones. In addition to these typical radiological images, sarcoidosis can present with a wide spectrum of atypical HRCT characteristics, such as unilateral lymphadenopathy in the parenchyma, which may manifest as a mass, ground-glass opacity with septal thickening, fibro-cystic changes, or even miliary distribution opacities [23,24].

Moreover, nodules can conglomerate and form larger tumor-like opacities when affecting the middle and upper lung fields. Thus, a tissue biopsy is the key to reaching a definitive diagnosis of sarcoidosis, as it confirmed the diagnosis in our case presented above.

As is well known, pulmonary sarcoidosis is part of interstitial lung diseases since, by definition, it involves an extensive inflammatory process of the pulmonary alveoli, small bronchi, and afferent blood vessels.

About 50% of patients require systemic treatment, while the remaining present spontaneous disease resolution. When a need for systemic therapy arises, clinicians typically administer prolonged treatment, and the likelihood of relapse is high upon its discontinuation. Systemic corticosteroids are the first-line treatment for sarcoidosis. Because of their many side effects, it is essential to consider other drugs in cases of intolerance to steroids (such as steroid-sparing agents) or in cases of the inefficiency of corticotherapy alone [25].

Another recommendation, in addition to symptomatology, is to consider the activity of granulomatous inflammation by using radiographic images of the lungs and checking the pulmonary function by performing spirometry and lung volume tests. Once these raise an alarming signal, immediate action is required with the introduction of corticosteroid therapy, although no pharmacological treatment has been approved for sarcoidosis yet. Demonstrations have shown that corticosteroids work in patients with sarcoidosis by inducing the regression of inflammatory genes such as interferon-gamma (IFN-y) and tumor necrosis factor (TNF)-alpha, which are the main cytokines identified in the development of necrotizing granulomas [26].

The typical outcome involves either natural resolution or improvement following systemic corticosteroid treatment. Nevertheless, there is a potential for relapses, even upon cessation of corticosteroid therapy [10].

## 4. Conclusions

Diagnosing pulmonary sarcoidosis becomes difficult due to the multiple forms of presentation of this multifaceted disease since, in some cases, its clinical and radiographic characteristics closely resemble those of a malignancy. Our objective was to highlight the exceptional origin of a lung mass and emphasize the significance of considering a wide range of potential diagnoses. However, given the potential for malignancy, maintaining a strong suspicion is crucial for prompt diagnosis and effective treatment.

## Data Availability

The data that support the findings of this study are available from the corresponding author upon reasonable request.
